# Request of hospital care dropped for TIA but remained stable for stroke during COVID-19 pandemic at a large Italian university hospital

**DOI:** 10.1007/s11739-020-02522-w

**Published:** 2020-10-15

**Authors:** Maurizio Balestrino, Alberto Coccia, Alessandra Silvia Boffa, Andrea Furgani, Francesco Bermano, Cinzia Finocchi, Monica Laura Bandettini di Poggio, Laura Malfatto, Daniele Farinini, Angelo Schenone

**Affiliations:** 1 Department of Neuroscience, Rehabilitation, Ophthalmology, Genetics and Mother and Child Sciences (DINOGMI), University of Genoa, Genoa, Italy; 2 IRCCS Ospedale Policlinico San Martino, Genoa, Italy; 3 Dipartimento Regionale di Emergenza Sanitaria Territoriale 118, Genoa, Italy

**Keywords:** COVID-19, Stroke, Transient ischemic attack, Ischemic stroke, Hemorrhagic stroke, Hospital care

## Abstract

Reduced incidence of stroke during COVID-19 pandemic was sometimes reported. While decrease in stroke incidence and fear of patients to go to the hospitals were sometimes invoked to explain this decrease, reduction in urban pollution was also hypothesized as a possible cause. We investigated statistically the incidence of ischemic and hemorrhagic stroke, and of transient ischemic attacks, at a large Italian tertiary stroke center during the pandemic. We analyzed statistically the number of transient ischemic attacks (TIA), ischemic strokes (IS) and hemorrhagic strokes (HS) between March 8 and May 2, 2020, the peak of the COVID-19 epidemic in Italy, and compared them with the identical period of 2019. We also analyzed the concentration of small particulate matter (PM_10_) in 2019 and 2020, to see if it could account for modified incidence of strokes or TIA. We found a large, significant drop in TIA (− 51%) during the pandemic compared to the same period of 2019. By contrast, the number of HS was identical, and IS showed a not significant − 24% decrease. PM_10_ concentration, already low in 2019, did not further decrease in 2020. Patients kept seeking hospital care when experiencing permanent neurological symptoms (stroke), but they tended not go to the hospital when their symptoms were transient (TIA). The fact that we did not observe a significant decrease in strokes may be explained by the fact that in our city the concentration of small particulate matter did not change compared to 2019.

## Introduction

Several papers reported decreased stroke incidence during the current COVID-19 pandemic [1–8]. Actual stroke reduction due to biological effects of the COVID-19 virus [9] and fear of patients to go to the hospital [7] have been invoked as possible causes of this decrease, but reduced air pollution has also been suggested as the possible cause of reduced stroke incidence during the 2020 pandemic [10]. Moreover, in the 2015 outbreak of Middle East Respiratory Syndrome (MERS), decrease of ischemic strokes was significant but limited (3.8–16.6%), and hemorrhagic strokes were unchanged [11]. We aimed at finding if and how the pandemic changed the incidence of ischemic and hemorrhagic strokes, and of transient ischemic attacks, at the Policlinic San Martino Hospital in Genoa, Italy, a regional “hub” and tertiary stroke care center. We also wanted to investigate if changes in air pollution during the epidemics could explain possible differences in stroke incidence.

## Methods

We analyzed statistically the observed numbers of transient ischemic attacks (TIA), ischemic strokes (IS) and hemorrhagic strokes (HS) at the Policlinic San Martino Hospital in Genoa, Italy, a regional “hub” and tertiary stroke care center. We considered as TIA all cases where neurological symptoms completely disappeared within 24 h. In addition to searching all cases that had been admitted to the Neurology ward or to the Stroke Unit, we also searched all cases where a neurological consultation had been done. The latter lead enabled us to detect even cases that had not been admitted to the Neurology ward nor to the Stroke Unit, as well as patients that had been discharged by the Emergency Room. We used the same definitions and search methods for 2019 and 2020, so whatever isolated cases might hypothetically have been missed, they were equally distributed between the two years. Genoa ranks sixth among Italian provinces for number of COVID-19 infections [12]. We investigated the 8 weeks (March 8–May 2) between the legislative acts that initiated and loosened the prohibition to exit home (“lockdown”) in Italy. They include rise and fall of the contagion curve in Italy, when the epidemics was most severe [12]. We compared this period with the identical period (March 8–May 2) of 2019, and we analyzed statistically the differences using the program Prism (GraphPad, USA). To gauge possible effects of changes in air pollution on the results we observed, we studied the concentrations of small particulate matter (PM_10_) within the catchment area of our hospital that were reported during the study periods by the relevant municipal agency [13].

## Results

During the study period, we observed 24 TIA (they had been 49 in 2019, − 51%); 79 IS (99 in 2019, − 20%); 20 HS (17 in 2019, + 15%). Since during the 2020 pandemic the “118 Liguria” agency (providing Emergency Ambulance Services in our region) were reorganized, ambulances brought to our hospital patients from outside its usual catchment area, too. Therefore, we risked to underestimate a possible decrease in strokes. Thus, we repeated the analysis considering only patients brought from the usual catchment area of our hospital. When doing so, the number of IS in 2020 became 75 (− 24% compared to 2019), and the number of HS became 17 (same as in 2019).

**Fig. 1:**
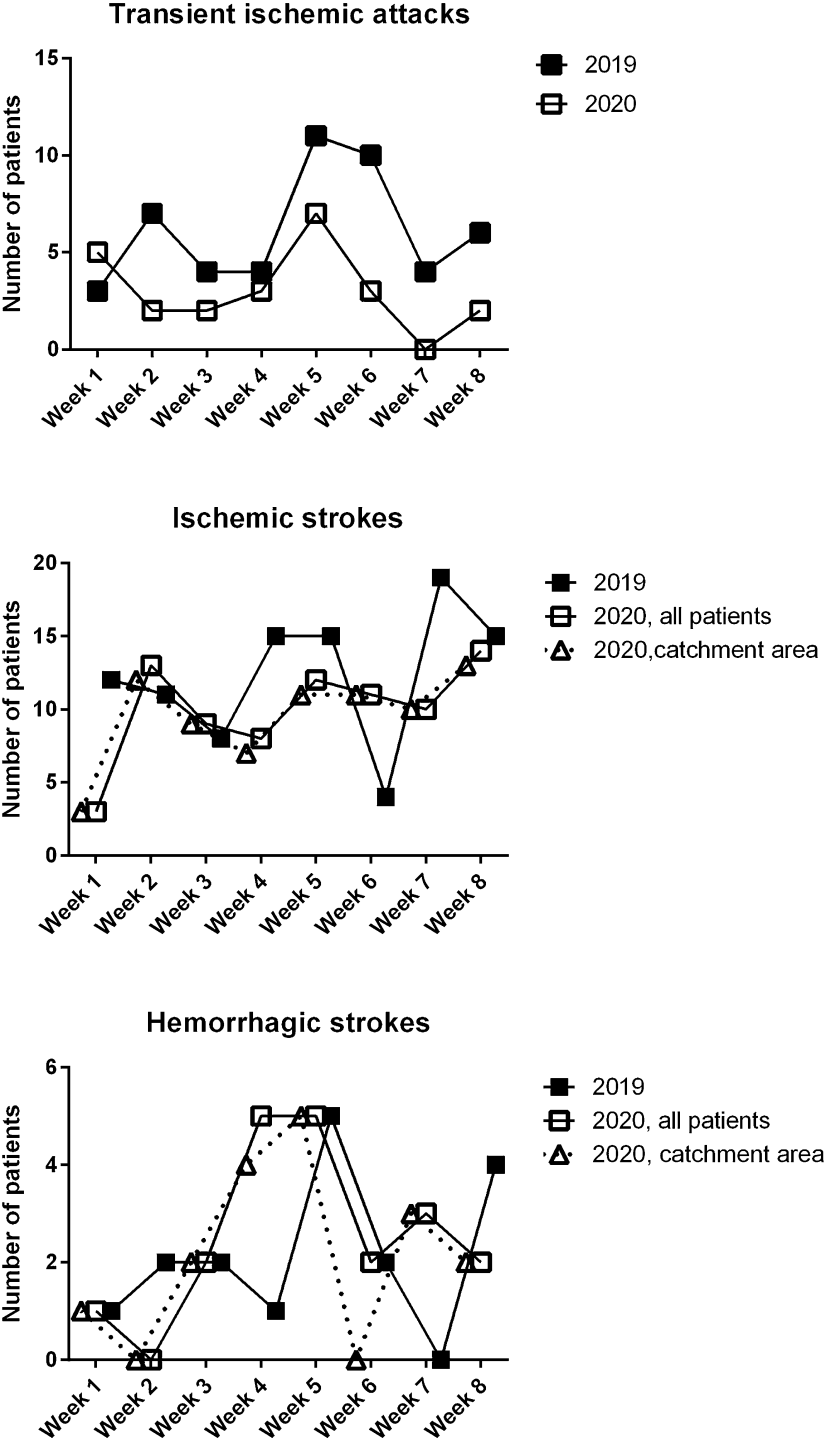
Weekly incidence of the various conditions. For 2020, we provide both the total numbers of ischemic and hemorrhagic strokes we observed, and the numbers of only those patients that came from within the usual catchment area of our hospital. Since TIA patients were not usually carried by ambulance, their number was unaffected by the reorganization of emergency services that took place in 2020. See text and Table 1 for further details

Table 1 summarizes the weekly incidence of the various conditions. Figure 1 depicts their occurrence in the single weeks.

**Table 1:** Statistical analysis of the rate of observation of the various conditions during the study periods.

	2019	2020 (all observed cases)	2020 (only patients from the hospital catchment area)	*p* value (*t* test)
Weekly transient ischemic attacks	6.1 ± 3.0	3.0 ± 2.1	N/A	0.03
Weekly ischemic strokes	12.0 ± 4.7	10.0 ± 3.5	9.5 ± 3.2	0.28; 0.20 (n.s.)
Weekly hemorrhagic strokes	2.1 ± 1.6	2.5 ± 1.8	2.1 ± 1.8	0.67; > 0.99 (n.s.)

Entries are mean ± standard deviation. In the last column, the first or only *p* value compares 2019 with all observed 2020 patients, the second one compares 2019 with 2020 patients from the catchment area of our hospital only.

## Discussion

Incidence of TIA dropped significantly across almost all the study period; however, we did not observe a comparable, significant decrease of ischemic strokes. In some weeks, IS were even increased compared to 2019 (Fig. 1). We interpret these findings as demonstrating the reluctance of patients experiencing transient symptoms to go to the hospital (in agreement with what was hypothesized by other authors) [6, 7], that in our experience contrasts with their maintained tendency to seek hospital care despite the pandemic when their symptoms were permanent (stroke). This drop in hospital evaluation of TIA is regrettable, because it likely impeded secondary stroke prevention, thus it is possible that a surge of IS might have happened afterwards. While we do not currently have data neither proving nor disproving the hypothesis of this surge, we think that in future epidemics a communication effort should be made to encourage people with even transient neurological symptoms to keep seeking neurological care. Other authors have also pointed out to the need to encourage hospital admission of non-Covid patients in possible future epidemics [14].

The number of HS did not change at all during the pandemic, a finding that was also reported in 2015 during the outbreak of MERS, another coronavirus epidemics [11]. The number of HS was small in both years, as it could have been expected since incidence of HS is usually only 10–20% of all strokes [15–17]. Nevertheless, the fact that we observed a virtually identical number of HS in 2019 and in 2020 seems to us worth considering. We interpret it as meaning that HS patients kept seeking hospital care in the same way in the 2020 as they had done in 2019.

As for IS, we found a not significant decrease in their occurrence during the epidemics. We cannot rule out that in a larger sample such a decrease might have resulted significant. However, we emphasize that IS decrease was statistically not significant even in the face of a significant and consistent drop in TIA (Table 1; Fig. 1). Moreover, it was half the magnitude of TIA decrease (− 24% for IS vs. − 51% for TIA). Thus, even if the pandemic-related decrease in IS incidence was actually real, in our city it was limited, and approximately of the same magnitude that was found during the outbreak of MERS in 2015 [11]. Finally, we emphasize that severity of strokes in 2020 was comparable to what had been in 2019, as shown by the National Institute of Health Stroke Scale and by the modified Rankin Scale at admission, that were both identical in the two periods. We are providing specific details of these scores in a further paper, now in preparation.

Finally, even if a true IS reduction was demonstrated, it should be explained by a selective reduction in events triggering ischemic, not hemorrhagic, events. Reduced air pollution has been suggested as a candidate for such an explanation [10]. We analyzed the concentration of small particulate matter (PM_10_) in our city during the study periods in 2019 and 2020. Specifically, we consulted the official data reported by the “Arpal” city agency [13], comparing the PM_10_ concentration in the measuring station of Corso Buenos Aires, which is located within the catchment area of our hospital. We found that its daily concentration was (mean ± standard deviation) 18 ± 5.4 μg/m^3^ in 2019, 19 ± 8.8 in 2020, a not significant difference (paired t test). Therefore, IS may have decreased less than in other areas because pollution, quite low to begin with, was not improved further by the lockdown, thus did not contribute to lower IS rate. Further research should be done to better understand the relationship between stroke and air pollution. However, the fact that we did not observe decrease in air pollution during the epidemics lockdown period suggests that this may be the reason why we did not observe the decrease in strokes that other authors reported.

Summing up, patients experiencing transient symptoms refrained from going to the hospital, possibly for fear of contagion. By contrast, patients showing permanent symptoms kept seeking hospital care. Among the latter ones, hemorrhagic strokes did not change at all compared to previous year, a finding that had already been observed in a previous similar epidemics [11]. Ischemic strokes showed a not significant decrease. Such lack of significant decrease may be explained at least in part by lack of change in air pollution, a stroke triggering factor that has been hypothesized as a cause for IS decrease during the pandemic [10].
